# Safety and Efficacy of Endovascular Treatment for Progressive Stroke in Patients With Acute Basilar Artery Occlusion

**DOI:** 10.3389/fneur.2021.774443

**Published:** 2021-12-16

**Authors:** Yinxu Wang, Yingbing Ke, Lingling Wang, Qing Wu, Jing Zhou, Xiaolin Tan, Jiazuo Liu, Wanjie Geng, Daoyou Cheng, Zongtao Liu, Yinquan Yu, Jiaxing Song, Zhongming Qiu, Fengli Li, Weidong Luo, Jie Yang, Wenjie Zi, Xiaoming Wang, Zhengzhou Yuan

**Affiliations:** ^1^Department of Rehabilitation Medicine, The First Affiliation: Jinan University, Guangzhou, China; ^2^Department of Rehabilitation Medicine, Affiliated Hospital of North Sichuan Medical College, Nanchong, China; ^3^Department of Neurology, Yangluo Branch of Hubei Zhongshan Hospital, Wuhan, China; ^4^Department of Neurology, Affiliated Hospital of North Sichuan Medical College, Nanchong, China; ^5^Department of Neurology, Meishan Second People's Hospital, Meishan, China; ^6^Department of Neurology, Bazhong Pingchang County People's Hospital, Bazhong, China; ^7^Department of Neurology, Anhui Provincial People's Hospital of Taihe County, Fuyang, China; ^8^Department of Neurology, Guizhou Xinyi People's Hospital, Xingyi, China; ^9^Department of Neurology, Anhui Province Taihe County Hospital of Traditional Chinese Medicine, Fuyang, China; ^10^Department of Neurology, Bazhong City Hospital of Traditional Chinese Medicine, Bazhong, China; ^11^Department of Neurology, Xinqiao Hospital and The Second Affiliated Hospital, Army Medical University (Third Military Medical University), Chongqing, China; ^12^Department of Neurology, Affiliated Hospital of Southwest Medical University, Luzhou, China

**Keywords:** basilar artery occlusion, progressive stroke, endovascular treatment, posterior circulation, time window

## Abstract

**Background and Purpose:** It is unknown the benefit of endovascular therapy (EVT) for progressive stroke in patients with basilar artery occlusion (BAO). The aim of this study was to compare the efficacy and safety of EVT with standard medical therapy (SMT) in a population of BAO patients with progressive stroke.

**Methods:** The EVT for Acute Basilar Artery Occlusion Study (BASILAR) is a national prospective registry of consecutive patients with acute BAO within 24 h of symptom onset. According to the applied therapy, all patients were divided into SMT and EVT groups. Subsequently, the EVT group was divided into early (≤6 h) and late groups (>6 h) according to the time window. The efficacy outcome was favorable functional outcomes (modified Rankin Scale score ≤ 3) at 90 days. The safety outcomes included mortality within 90 days and symptomatic intracerebral hemorrhage (sICH) after EVT.

**Results:** The EVT cohort presented more frequently with a favorable functional outcome (adjusted odds ratio, 5.49; 95% confidence interval, 2.06–14.61, *p* = 0.01) and with a decreased mortality (adjusted odds ratio, 0.3; 95% confidence interval, 0.17–0.54, p < 0.001). What's more, EVT still safe (*P* = 0.584, *P* = 0.492, respectively) and effective (*P* = 0.05) in patients with progressive stroke when the treatment time window exceeds 6 h.

**Conclusions:** EVT was more effective and safer than SMT for progressive stroke in patients with BAO. Besides, EVT remains safe and effective in patients with progressive stroke when the treatment time window exceeds 6 h. Predictors of desirable outcome in progressive stroke patients undergoing EVT included lower baseline NIHSS score, higher baseline pc-ASPECTs, successful recanalization and shorter puncture to recanalization time.

## Introduction

Posterior circulation stroke has a high mortality and disability rate, especially when caused by basilar artery occlusion (BAO) (1). Recently, several prospective studies have showed that patients with BAO treated with endovascular therapy (EVT) may have better functional outcomes than those treated with standard medical therapy (SMT) alone (2, 3). Moreover, pooled estimates of successful recanalization can reach 80%. One meta-analysis demonstrated that 42% of patients with BAO to achieve functional independence after stent retriever thrombectomy (4). The results of a number of studies show that in clinical practice, EVT is an effective and safe therapy for BAO.

Progressive stroke is defined as a progressive or gradual worsening of neurological function after the onset of ischemic stroke that persists over time until more severe neurological deficits appear (5). At the beginning of the disease, there are only prodromal symptoms such as dizziness, headache, and mild numbness and weakness of the limbs. There is no international definition of progressive stroke and most studies suggest that it is four points higher than the admission or baseline values on the National Institutes of Health Stroke Scale (NHISS) (6). The incidence of progressive stroke as defined by the above criteria ranges from 13.8 to 17.6% (7, 8). Recent studies have shown the effectiveness and safety of EVT in patients with progressive stroke due to large vessel occlusion in the anterior circulation. EVT has a better outcome than SMT without increasing the incidence of adverse events (9, 10). And although EVT has been used in the past few years to treat BAO with a trend toward benefit, there are limited data on the efficacy and safety of EVT in progressive stroke. In addition, as mentioned earlier the initial neurological status of progressive stroke is mild. When the neurological deficit is moderate or severe, the time window often exceeds 6 h. A study has shown that EVT is a safe treatment option for patients with progressive stroke due to anterior circulation infarction, even beyond the 6-h time window (9). However, the clinical outcome of EVT in patients with progressive stroke in acute BAO beyond 6 h is unknown.

Here, based on the largest multi-center consecutive BAO cohort to date, this study will be the first to compare the efficacy and safety of EVT and SMT in progressive stroke, and analyze the clinical outcomes of EVT in patients with progressive stroke beyond the 6-h time window.

## Methods

The data supporting the results of this study can be obtained from the corresponding author upon reasonable request.

### Patients Selection

Patients with symptomatically and radiologically confirmed acute BAO within 24 days in 47 comprehensive stroke centers in China between January 2014 and May 2019 were selected for this study 错误!未找到引用源。(11). Patients were divided into two groups according to their primary treatment modality: either SMT alone (SMT group) or SMT plus EVT (EVT group). The inclusion criteria for this study met the following criteria: (1) age ≥ 18 years; (2) presentation within 24 h of estimated time of BAO; (3) a BAO confirmed by computed tomography angiography, magnetic resonance angiography, or digital subtraction angiography; (4) informed consent, and (5) meeting the definition of progressive stroke, it is four points higher than the admission or baseline values on the National Institutes of Health Stroke Scale (NHISS). Patients were excluded from the study in the case of (1) a premorbid modified Rankin Scale (mRS) score >2; (2) evidence of intracranial hemorrhage on presentation; (3) a lack of follow-up information; (4) current pregnancy or lactation; (5) a serious, advanced, or terminal illness; and (6) incomplete baseline critical data (e.g., imaging and time metrics).

All the participating centers were approved by the ethics committee, and informed consent was obtained for all enrolled patients. BASILAR was registered with the Chinese Clinical Trial Registry (http://www.chictr.org.cn, ChiCTR1800014759). The authors have stated explicitly that there are no conflicts of interest in connection with this article.

### Endovascular Treatment

The choice of treatment is based on a comprehensive assessment by the treating physician and the wishes of the patient or his or her legal representative. Patients are treated according to guidelines for the management of acute ischemic stroke, such as intravenous thrombolysis with recombinant tissue fibrinogen activator within 4.5 h and intravenous thrombolysis with urokinase within 6 h of the estimated time for various combinations of antiplatelet agents, systemic anticoagulation, and other methods. If it is confirmed on the CTA that the patient has BAO corresponding to stroke symptoms, EVT such as thrombus contraction, aspiration, or the use of a stent recovery device are adopted. If recanalization fails firstly, salvage therapy such as balloon dilation and stent placement may be used. Recanalization was assessed using the Thrombolysis in Cerebral Infarction Inventory (mTICI), with successful recanalization defined as having a mTICI of 2b/3 flow (12).

### Parameter Definitions

The BASILAR cohort was divided into a progressive stroke group and a non-progressive stroke group. Our current study population was progressive stroke, which was divided into the SMT group (including antiplatelet or anticoagulation therapy, intravenous thrombolysis, or a combination of these therapies) and the EVT group (standard medications plus mechanical embolization, thrombus aspiration, balloon angioplasty, stent placement, or a combination of these methods) based on treatment modality. The specific treatment is at the discretion of the local physician. In the second step, the EVT cohort was divided into the Early group (within 6 h) and the Late group (beyond 6 h) according to the treatment time window.

To evaluate the possible causative mechanisms of stroke according to the ORG10172 trial in the Acute Stroke Treatment (TOAST) Classification (13). The ischemic changes were quantified by the Posterior Circulation Alberta Stroke Program Early Computed Tomography Score (pc-ASPECTS, range 0–10, score≥8 correlated with the favorable outcome) (14). Collateral circulation status was assessed by the posterior circulation collateral score (PC-CS), which is based on the presence of potential collateral pathways in computed tomography angiography (15). Successful revascularization was defined as modified thrombolysis with a cerebral infarction score of 2b or three at the end of the procedure as confirmed by the imaging core laboratory based on individual angiography data (16).

### Study Outcomes

The efficacy outcome was a favorable functional outcome, defined as a proportion modified Rankin Scale score (mRS) ≤3 at 90 days. mRS is a seven-level classification scale used to measure functional outcome, ranging from 0 (asymptomatic) to 6 (death). The safety outcomes included the incidence of death within 90 days and the incidence of symptomatic intracranial hemorrhage (sICH) diagnosed within 48 h of EVT, confirmed by CT scan or magnetic resonance imaging. In addition, some procedure-related complications and serious adverse events were also analyzed.

### Statistical Analysis

Statistical analyses were performed using SPSS version 26 (IBM Corp, Armonk, NY, USA) and STATA version 16 (Stata Corp LLC, TX). A 2-tailed *P* < 0.05 was considered statistically significant differences. Continuous variables were expressed as mean with SD or median with interquartile range, while categorical data were expressed as counts and percentages. Univariate comparisons were performed using Fisher exact test or χ2 test for categorical variables, Kruskal Wallis test or Mann–Whitney U test for continuous variables.

Binary logistic regression models were used to assess the treatment effect of EVT and to identify predictors of functional outcome and mortality. Adjusted and unadjusted dominance ratios (ORs) were reported with 95% confidence intervals (CIs) to indicate statistical precision. Percentage bar graphs were plotted using Excel 2020 software (Microsoft). The adjusted margin plot shows the correlation between puncture to recanalization time (PTR) and favorable outcomes at 90 days. Distribution surfaces representing changes in the probability of predicted outcomes were generated using SigmaPlot 12.5. In addition, forest plots were analyzed for the probability of favorable function outcomes at 90 days in all predefined subgroups, including age, sex, baseline NHISS score, baseline pc-ASPECTES, PC-CS score, intravenous thrombolysis (IVT), and onset to imaging diagnosis time.

## Results

### Baseline Characteristics

Eight hundred and twenty-nine acute BAO patients from 47 stroke sites in China were enrolled in the BASILAR registry. Of these, there were 407 patients were progressive stroke [74.4% male; median (IQR) age, 63 (55–71) years]. When dichotomizing the progressive stroke patients, 312 of 407 (76.66%) were EVT cohort [median (IQR) age, 63 (54.25–71) years] and 95 of 407 (23.34%) were SMT cohort [median (IQR) age, 66 (59–74) years]. When the EVT cohort was dichotomized, 171 of the 312 (54.8%) patients were in the early group [median (IQR) age, 63 (55–71) years] and 141 (45.2%) were in the late group [median (IQR) age, 62 (55–70.25) years]. The baseline characteristics of the patients with progressive stroke and univariate analysis are presented in Table 1. Patients receiving EVT were younger, had higher baseline ASPECTs, and lower PC-CS score.

**Table 1:** Baseline characteristics of patients with progressive stroke.

	**All patients,** ***n* = 407**	**SMT,** ***n* = 95**	**SMT+EVT,** ***n* = 312**	***P*-value**	**EVT**		
					**Early group,** ***n* = 171**	**Late group,** ***n* = 141**	***P*-value**
Age–median (IQR)	63(55–71)	66(59–74)	63(54.25–71)	0.003	63(55–71)	62(54–70)	0.815
Sex, *n* (%)				0.204			0.812
Male	303(74.4)	66(69.5)	237(76)		129(75.4)	108(76.6)	
Female	104(25.6)	29(30.5)	75(24)		42(24.6)	33(23.4)	
NIHSS baseline score–median (IQR)	25(17–33)	24(17–32)	26(18–33)	0.456	27(16–34)	25(18–32)	0.794
pc-ASPECTs baseline-media (IQR)	8(6–9)	7(6–8)	8(6–9)	<0.001	8(7–9)	8(6–9)	0.029
PC-CS score-media (IQR)	4(3–6)	5(3–7)	4(3–6)	0.02	4(2–6)	4(3–6)	0.471
Pre-stroke mRS, *n* (%)				0.596			0.912
0	349(85.7)	79(83.2)	270(86.5)		147(86)	123(87.2)	
1	36(8.8)	9(9.5)	27(8.7)		15(8.8)	12(8.5)	
2	22(5.4)	7(7.4)	15(4.8)		9(5.3)	6(4.3)	
Medical history, *n* (%)							
Ischemic stroke	106(26)	29(30.5)	77(24.7)	0.256	42(24.6)	35(24.8)	0.958
Hypertension	289(71)	72(75.8)	217(69.6)	0.241	120(70.2)	97(68.8)	0.792
Diabetes mellitus	88(21.6)	21(22.1)	67(21.5)	0.896	37(21.6)	30(21.3)	0.938
coronary heart disease	53(13)	15(15.8)	38(12.2)	0.36	23(13.5)	15(10.6)	0.45
Atrial fibrillation	52(12.8)	8(8.4)	44(14.1)	0.146	27(15.8)	17(12.1)	0.346
Hyperlipidemia	139(34.2)	36(37.9)	103(33)	0.38	61(35.7)	42(29.8)	0.271
Cause of stroke, *n* (%)				0.28			0.105
Large artery atherosclerosis	306(75.2)	76(80)	230(73.7)		119(69.6)	111(78.7)	
Cardioembolism	69(17)	11(11.6)	58(18.6)		39(22.8)	19(13.5)	
Other causes	32(7.9)	8(8.4)	24(7.7)		13(7.6)	11(7.8)	
Location of occlusion, *n* (%)				<0.001			0.003
Basilar artery distal	87(21.4)	8(8.4)	79(25.3)		57(33.3)	22(15.6)	
Basilar artery middle	160(39.3)	66(69.5)	94(30.1)		46(26.9)	48(34)	
Basilar artery proximal	70(17.2)	8(8.4)	62(19.9)		27(15.8)	35(24.8)	
Vertebral artery-V4segement	90(22.1)	13(13.7)	77(24.7)		41(24)	36(25.5)	
Type of endovascular treatment							0.95^a^
Stent retriever thrombectomy	NA	NA	235(76.1)		131(77.5)	104(74.3)	
Aspiration	NA	NA	7(2.3)		3(1.8)	4(2.9)	
Ballon angioplasty and/or stenting	NA	NA	40(12.9)		19(11.2)	21(15)	
Intra-arterial medication and/or mechanical fragmentation	NA	NA	27(8.7)		16(9.5)	11(7.8)	
Time metrics, min, median (IQR)							
Onset to imaging diagnosis	200(68–379)	233(75–380)	196(64.75–371)	0.653	108(31–186)	390(246–551)	<0.001
Onset to treatment	240(108–406)	262(101–419)	238.5(108.25–403)	0.796	NA	NA	NA
Puncture to recanalization	NA	NA	107(75–154.75)	NA	NA	NA	NA
Procedural-related complications, *n* (%)							
Arterial perforation	NA	0	5(1.6)	NA	5(2.9)	0	0.067^a^
Arterial dissection	NA	0	2(0.6)	NA	1(0.6)	1(0.7)	1^a^
Distal embolization	NA	0	11(3.5)	NA	4(2.3)	7(5)	0.231^a^
Severe adverse events, n (%)							
Pulmonary infection	310(76.2)	77(81.1)	233(74.7)	0.202	118(69)	115(81.6)	0.011
Respiratory failure	155(38.1)	33(34.7)	122(39.1)	0.443	60(35.1)	62(44)	0.11
Circulatory failure	88(21.6)	19(20)	69(22.1)	0.661	30(17.5)	39(27.7)	0.032
Ulcer	79(19.4)	21(22.1)	58(18.6)	0.448	25(14.6)	33(23.4)	0.047
Venous thrombosis	22(5.4)	2(2.1)	20(6.4)	0.104	14(8.2)	6(4.3)	0.158
mTICI score 2b/3, *n* (%)	253(62.2)	3(3.2)	250(80.1)	<0.001	136(79.5)	114(80.9)	0.771

a *The P-values were estimated from Fisher precision inspection*. *IQR, interquartile rage; EVT, endovascular therapy; SMT, standard medical therapy; NIHSS, National Institutes of Health Stroke Scale; pc-ASPECTS, posterior circulation Alberta Stroke Program Early Computed Tomography Score; PC-CS score, posterior circulation collateral system score; mRS, modified Rankin scale scores; mTICI, modified Thrombolysis in Cerebral Infarction. Earel group, onset to puncture time ≤ 360 min (6 h); Late group, onset to puncture time > 360 min (6 h)*.

### Outcomes of EVT vs. SMT

**Figure 1:**
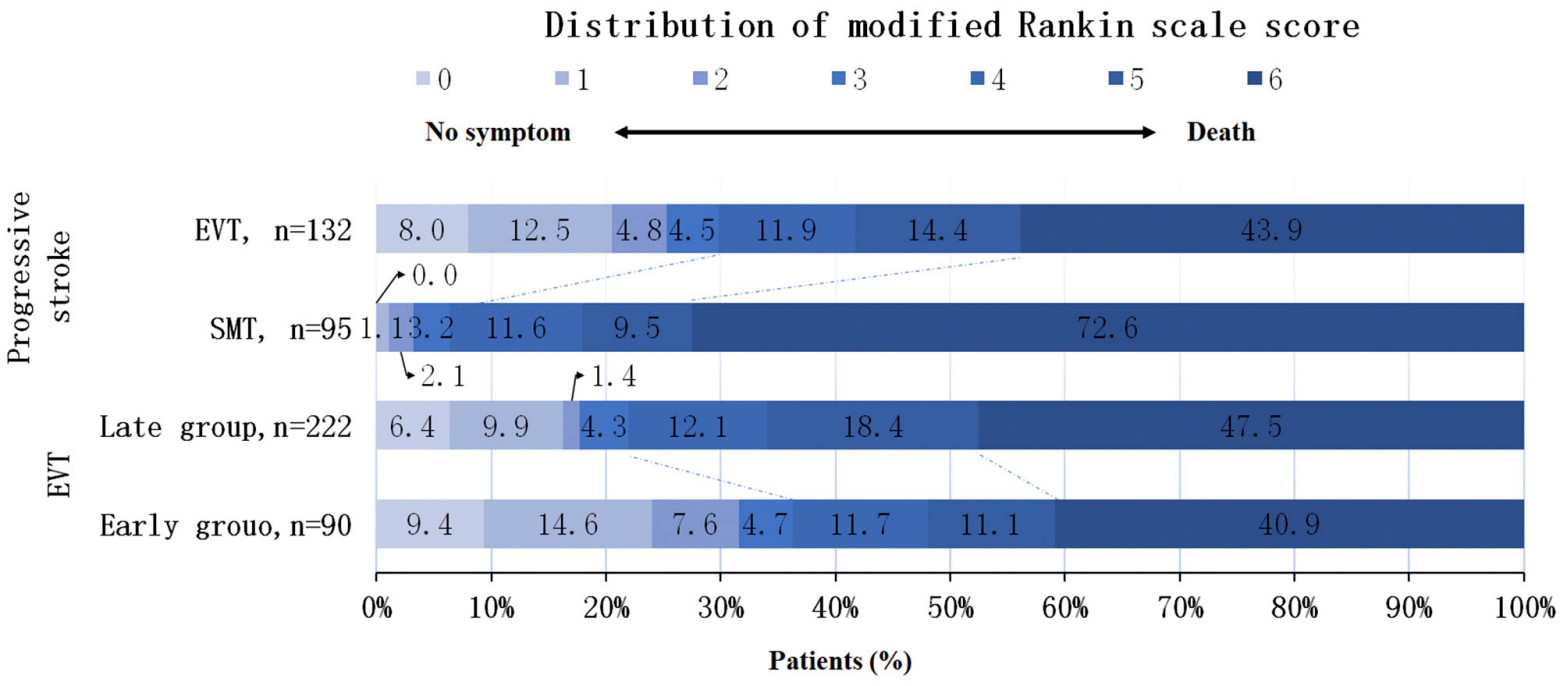
Distribution of modified Rankin Scale scores at 90 days in a population with progressive stroke. Shown was the distribution of the modified Rankin scale scores at 90 days in patients with progressive stroke. The distribution shows that EVT are associated with higher rates of favorable functional outcomes and lower mortality in patients with progressive stroke compared to SMT. In the EVT cohort, the incidence of favorable functional outcome was higher in the early group, but there was no significant difference in mortality. EVT, Endovascular therapy; SMT, standard medical therapy.

In the univariate analysis, the EVT cohort showed a higher rate of favorable functional outcome (29.8 vs. 6.3%; *p* < 0.001) and a reduced mortality (43.9 vs. 72.6%; *p* < 0.001) compared with the SMT cohort (Figure 1). Even when defined by different mRS scores, the functional outcomes of the EVT cohort were better than those of the SMT cohort (mRS 0–2: 25.3 vs. 3.2%, *p* < 0.001; mRS 0–1: 20.5 vs. 1.1%, *p* < 0.001). The multivariate analysis confirmed that the favorable functional outcomes (mRS ≤ 3) [adjusted odds ratio with 95% CI, 6.68 (2.58–17.34)] still favoring EVT. And the mortality rate was lower [adjusted odds ratio with 95% CI, 0.26 (0.15–0.47)]. Although the incidence of sICH was higher in the EVT group than in the SMT group, the difference was not statistically significant (*P* = 0.091) (Table 2).

**Table 2:** Efficacy and Safety Outcomes.

	**SMT vs. EVT**				**Early group vs. Late group**			
	**Unadjusted** **OR (95% CI)**	***P*-value**	**Adjused odds** **ratio (95% CI)^a^**	***P*-value^b^**	**Unadjusted** **OR (95% CI)**	***P*-value**	**Adjused odds** **ratio (95% CI)^c^**	***P*-value^b^**
**Efficacy outcome**								
mRS score 0–3 at 90 d, *n* (%)	6.3 (2.66–14.91)	<0.001	6.68 (2.58–17.34)	<0.001	0.5(0.3–0.82)	0.007	0.46 (0.21–0.999)	0.05
**Safety outcomes**								
Mortality at 90 d, *n* (%)	0.3 (0.18–0.49)	<0.001	0.26 (0.15–0.47)	<0.001	1.31 (0.83–2.05)	0.244	1.19 (0.64–2.24)	0.584
SICH, *n* (%)	5.76 (0.76–43.69)	0.091	4.53 (0.58–35.6)	0.149	0.97 (0.37–2.52)	0.948	1.51 (0.47–4.85)	0.492

a *The multiple logistic regression test was used to analyze ORs. Adjusted variables: age, NIHSSbaseline, PCCSScore, ASPECTSbaseline, occlusion site, Onset-Imaging Time*.

b *The Bonferroni correction method was applied to multiple comparisons using a p-value < 0.05/number of comparisons as a threshold for statistical significance*.

c *The multiple logistic regression test was used to analyze ORs. Adjusted variables: NIHSSbaseline, ASPECTSbaseline, occlusion site, Onset-Imaging Time. CI, confidence interval; mRS, modified Rankin Scale; SICH, symptomatic intracranial hemorrhage*.

### Outcomes of Early Group vs. Late Group in EVT Cohort

**Figure 2:**
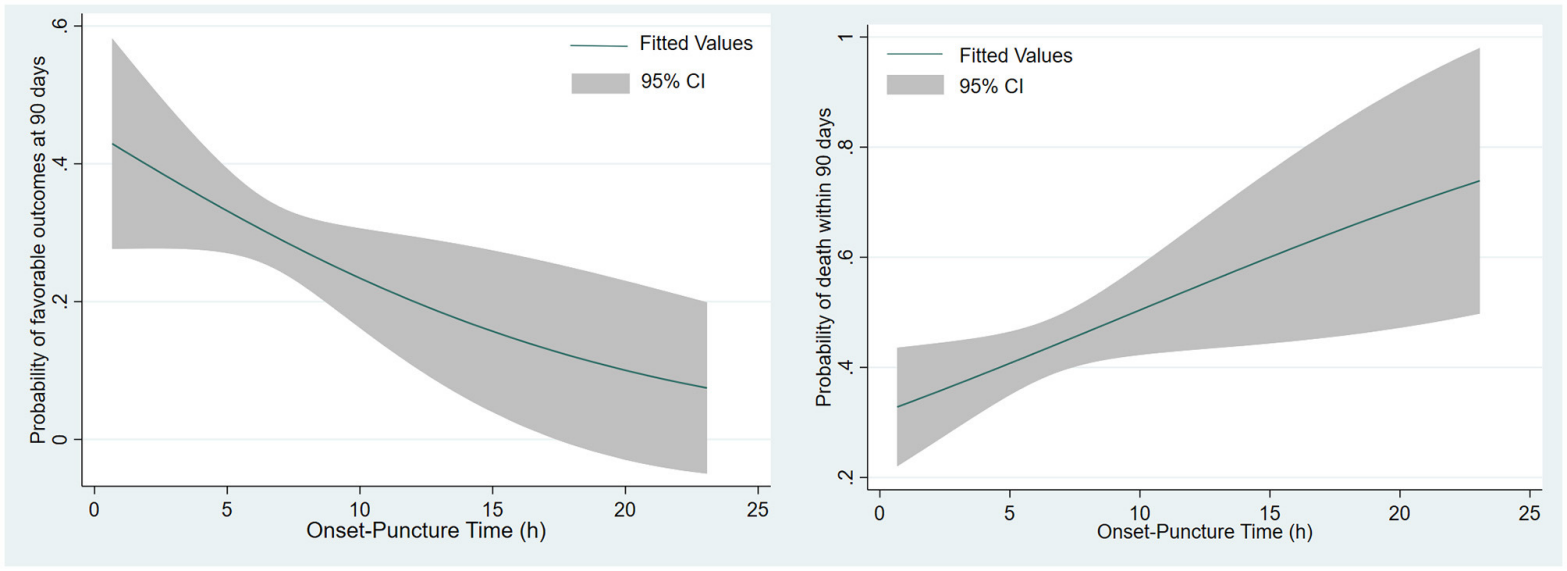
Probability of predicting clinical outcomes by onset to puncture time in EVT cohort. In all patients receiving the intervention, the curves show that the predicted probability of a good functional outcome decreases with increasing episode to puncture and the predicted probability of death increases.

In univariate analysis, the early group showed a higher rate of favorable functional outcome compared to the late group (36.3 vs. 22%; *p* = 0.007), but no significant difference in mortality (40.9 vs. 47.5%; *p* = 0.584) (Figure 1). Furthermore, multivariate analysis confirmed that although the favorable functional outcome (mRS ≤ 3), mortality and sICH differed between the two groups, the differences were not statistically significant [adjusted odds ratio with 95% CI, 0.46 (0.21–0.999), 1.19 (0.64–2.24), 1.51 (0.47–4.85), respectively] (Table 2). In the EVT cohort, as the time from onset to puncture (OTP) increased, the probability of favorable functional outcomes decreased and death within 90 days increased (Figure 2).

### Predictors of Outcome With EVT in Patients With Progressive Stroke

**Figure 3:**
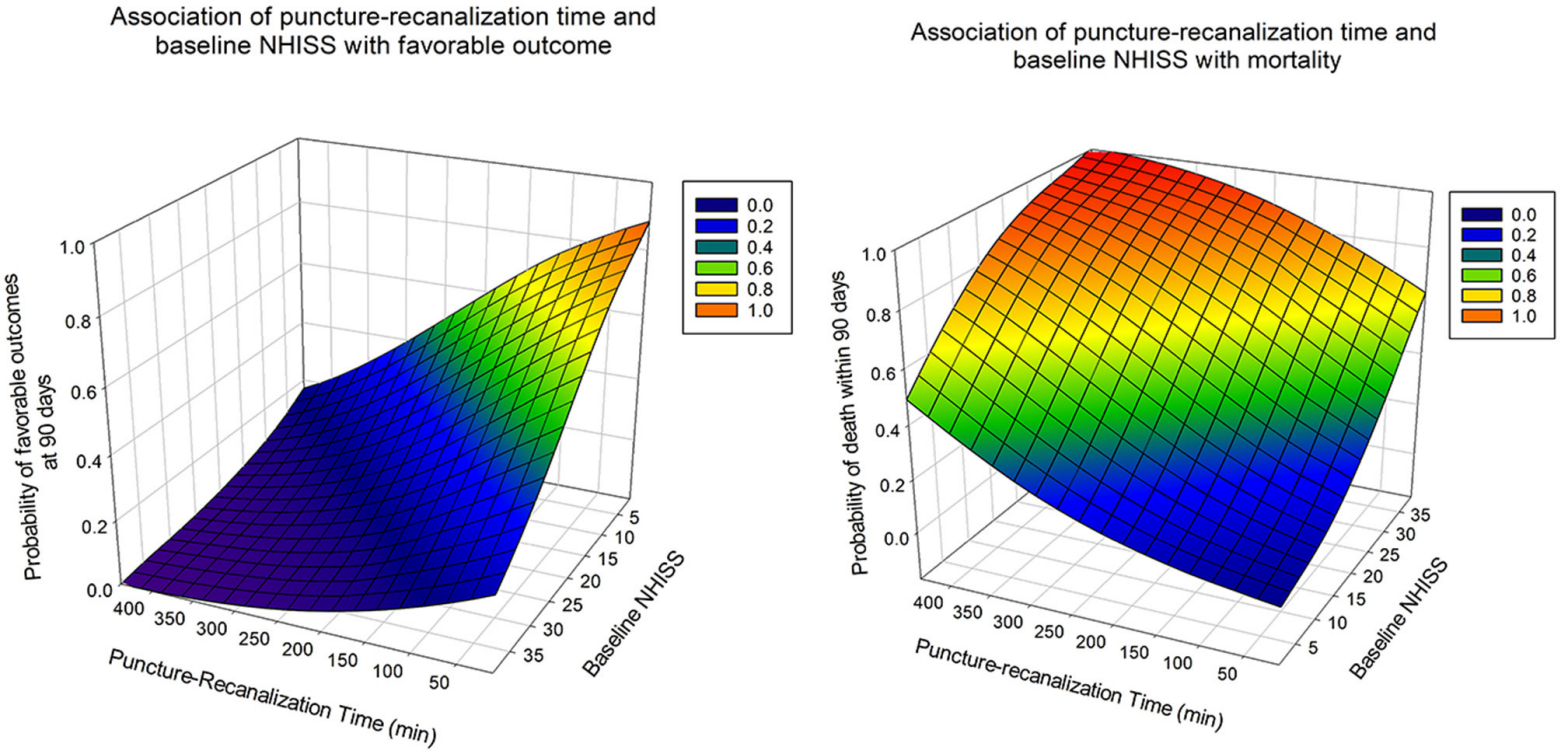
Association of puncture to recanalization time and baseline NIHSS with the probability of good functional outcome at 90 days (left) and the probability of death within 90 days (right) in patients with progressive stroke who received the intervention.

Predictors of prognosis after receiving EVT were further explored in the current study, in which we dichotomized 312 patients with progressive stroke who received the intervention by functional outcome (favorable vs. unfavorable) and mortality (alive vs. death) to determine the variables to be adjusted. The results showed that baseline NIHSS (OR, 0.92; 95% CI, 0.89–0.95; *p* < 0.001), baseline pc-ASPECTs (OR, 1.69; 95% CI, 1.37–2.07; *p* < 0.001), puncture-recanalization time (OR, 0.99; 95% CI, 0.99–0.999; *p* = 0.016), and successful recanalization (OR, 3.97; 95% CI, 1.57–10; *p* = 0.003), as predictors of favorable functional outcomes. Baseline NIHSS (OR, 1.08; 95% CI, 1.04–1.11; *p* < 0.001), PC-CS score (OR, 0.84; 95% CI, 0.73–0.97; *p* = 0.016), baseline pc-ASPECTs (OR, 0.73; 95% CI, 0.62–0.87; *p* < 0.001), puncture-recanalization time (OR, 1.01; 95% CI, 1–1.02; *p* < 0.001), and mTICI (OR, 0.08; 95% CI, 0.04–0.18; *p* < 0.001) were predictors of mortality (Table 3). The predicted outcome probabilities for PTR and baseline NHISS are shown by 3D plots (Figure 3).

**Table 3:** Predictors of outcome following EVT in progressive stroke patients.

	**Unadjusted OR (95% CI)**	***P*-value**	**Adjusted OR (95% CI)^a^**	***P*-value**
**Favorable functional outcome**				
Male	1.12(0.63–2.0)	0.695		
Age	0.98(0.96–1.001)	0.058		
Baseline NIHSS	0.92(0.89–0.94)	<0.001	0.92(0.89–0.95)	<0.001
Baseline ASPECTs	1.72(1.43–2.07)	<0.001	1.69(1.37–2.07)	<0.001
PC-CS score	1.18(1.05–1.33)	0.007	1.08(0.94–1.25)	0.285
Location of occlusion				
Distal BA^b^		0.059		
Middle BA	2.35(1.18–4.7)	0.015		
Proximal BA	1.25(0.63–2.51)	0.524		
VA-V4	1.14(0.53–2.48)	0.741		
TOAST				
Large artery atherosclerosis		0.055		
Cardioembolism	0.59(0.25–1.41)	0.236		
Other causes	1.18(0.44–3.13)	0.745		
Puncture-recanalization time	0.995(0.99–0.999)	0.023	0.993(0.99–0.999)	0.016
mTICI, 2b/3	4.12(1.8–9.44)	0.001	3.97(1.57–10)	0.003
**Mortality**				
Male	0.995(0.59–1.68)	0.986		
Age	1.02(0.997–1.04)	0.102		
Baseline NIHSS	1.08(1.05–1.11)	<0.001	1.08(1.04–1.11)	<0.001
Baseline ASPECTs	0.71(0.61–0.82)	<0.001	0.73(0.62–0.87)	<0.001
PC-CS score	0.83(0.74–0.93)	0.001	0.84(0.73–0.97)	0.016
TOAST				
Large artery atherosclerosis		0.102		
Cardioembolism	2.19(0.87–5.48)	0.094		
Other causes	1.38(0.29–3.86)	0.542		
Puncture-recanalization time	1.01(1.003–1.011)	0.001	1.01(1–1.02)	<0.001
mTICI, 2b/3	0.11(0.06–0.23)	<0.001	0.08(0.04–0.18)	<0.001

a *Adjusted estimates of effect were calculated using multiple regression taking the following variables into account: baseline NIHSS, baseline pc-ASPECTs, PC-CS score, puncture-recanalization time, mTICI*.

b *Distal basilar artery was taken as reference*. *CI, confidence interval; NIHSS, National Institutes of Health Stroke Scale; OR, odds ratio; PC-CS score, posterior circulation collateral system score; pc-ASPECTS, posterior circulation Alberta Stroke Program Early Computed Tomography Score; EVT, endovascular therapy; mTICI, modified Thrombolysis in Cerebral Infarction*.

### Subgroup Analyses

**Figure 4:**
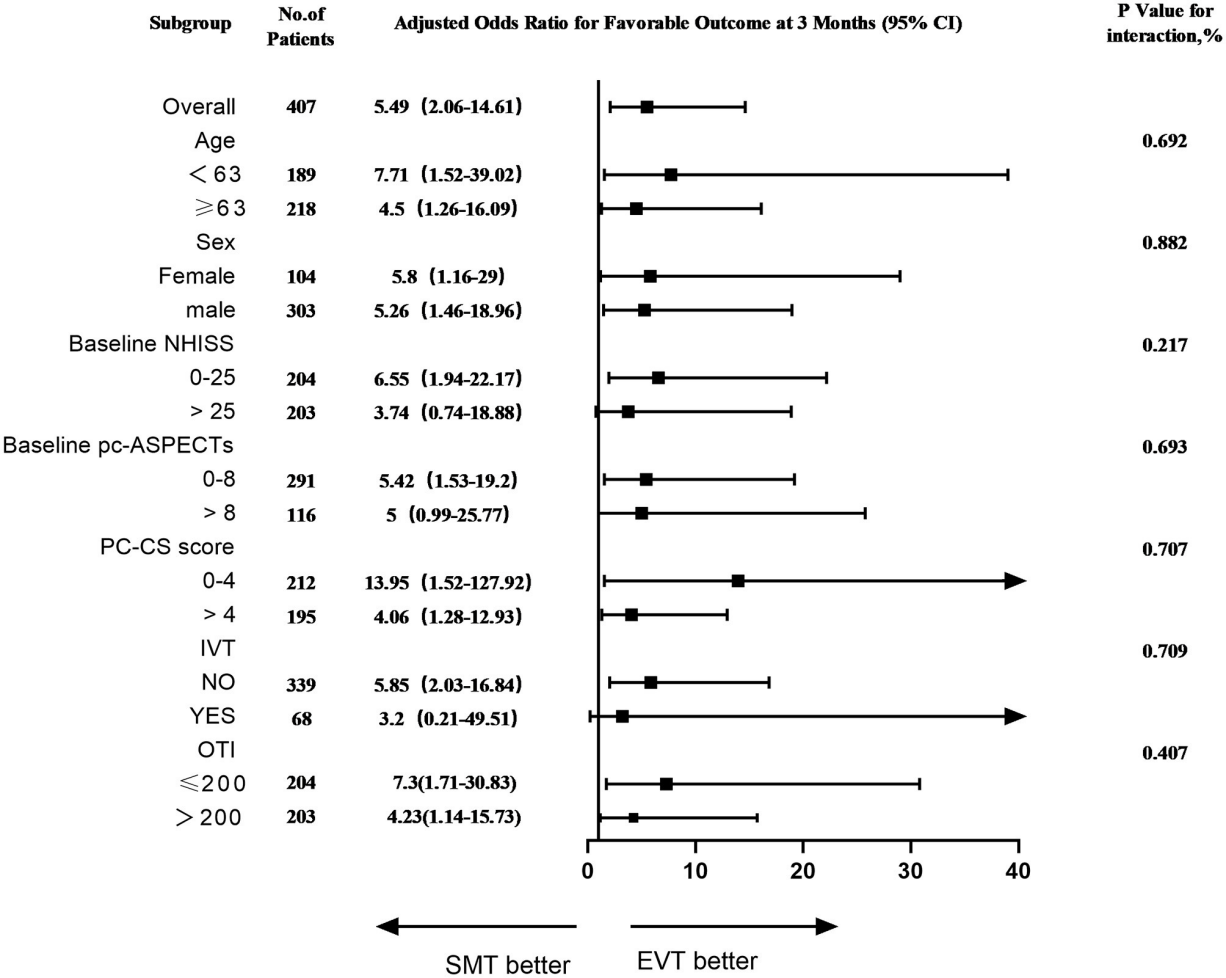
Subgroup analyses of primary outcomes. The forest plot shows the differences in odds ratios for favorable outcomes (defined as modified Rankin Scale score of 0–3) at 3 months in the prespecified subgroups. Adjusted variables: age, National Institutes of Health Stroke Scale (NIHSS), onset to imaging time, occlusion site, posterior circulation collateral system score (PC-CS), posterior circulation Alberta Stroke Program Early CT Score (pc-ASPECTS). IVT, intravenous thrombolysis; OTI, onset to imaging diagnosis time.

Based on the progressive stroke population, this forest plot shows that the probability of favorable functional outcomes at 90 days in all of predefined subgroups, including age, sex, baseline NHISS, baseline pc-ASPECTES, PC-CS score, IVT and onset to imaging diagnosis time (OTI). Overall, the EVT group was more likely to have a favorable functional outcome than the SMT group [adjusted OR, 5.49 [95% CI, 2.06–14.61)]. What' more, it's more likely for EVT group to have favorable functional outcomes than SMT group in all subgroups for age, sex, PC-CS score, and OTI (Figure 4).

## Discussion

To our knowledge, this multicenter cohort study is the first to compare the efficacy and safety of EVT and SMT in patients with progressive stroke in acute BAO. The main finding of the study was the strong correlation between EVT and favorable outcome and safety in progressive stroke. And EVT is safe and effective in patients with progressive stroke even when the treatment time window exceeds 6 h. What's more, the recanalization rate (mTICI ≥ 2b) was 80.1% in the EVT group. And there was no statistical difference in the incidence of adverse events between the SMT and EVT group. The findings provide extensive evidence for the choice of EVT for patients with progressive stroke in acute BAO and for clinicians.

The published literature reports outcomes of endovascular therapy in patients with progressive stroke in the anterior circulation compared with standard drug therapy. In trials of endovascular therapy for progressive ischemic stroke in anterior circulation large artery occlusion, the overall outcome of EVT was significantly better than drug therapy, even beyond the traditional 24-h time window, and endovascular therapy may improve neurological recovery within 90 days (9, 10). Relatively few studies have been reported on patients with progressive stroke in BAO. Many studies have confirmed the efficacy and safety of EVT after intervention in patients with acute BAO (1–4), and individual studies have reported adverse regression rates of EVT in BAO patients ranging from 54 to 95% (17). However, few studies have focused only on the progressive stroke population in acute BAO. Our results clearly demonstrate the efficacy and safety of EVT in patients with progressive stroke. Of the 407 subjects enrolled in the trial, 76.66% were in the EVT cohort and 23.34% were in the SMT cohort. Vascular risk factors and baseline NHISS were well matched in both groups (*P* > 0.05). Despite differences in pc-ASPECTs score and PC-CS score between the two groups, the EVT group still had a significantly better rate of favorable functional outcome and death than the SMT group after multifactorial analysis. Therefore, this study is a guideline for the treatment of patients with progressive stroke in acute BAO by studying the progressive stroke population.

Our study also compared for the first time the effect of EVT on patients with progressive stroke with a treatment time window of more than 6 h. The result showed that EVT is safe to take in patients with progressive stroke in acute BAO with a time window of more than 6 h. Although the time from onset to diagnosis was significantly longer in the late group than in the early group, EVT application was still shown to be safe and effective in the late group after multifactorial analysis. Previous studies have found that despite the poor prognosis of progressive ischemic stroke due to proximal large artery occlusion (18), they also confirmed the safety of endovascular therapy in the late group of patients with progressive stroke (9). Although the time window for endovascular treatment of ischemic stroke has been extended to 24 h (12), 6 h is still often used as a reference in clinical practice. Our study, with progressive stroke as the target population and a 6-h cut-off, provides clinical staff with more diverse and reliable data to support the use of EVT in acute BAO-induced progressive stroke.

In addition, clinical outcomes were better in patients with progressive stroke who received EVT intervention. Multiple logistic regression analysis showed that low baseline NIHSS score, high baseline pc-ASPECTs, good collateral status, successful recanalization and short operative time were all associated with desirable outcomes, consistent with previous studies (19–22). However, there are fewer reports on the duration of the procedure, so we focused on the prognostic impact of the time from puncture to recanalization on EVT. Recently, many articles have reported the influence of procedure time on the results of EVT, showing that as the procedure time increases, the functional independence is lower (23, 24). Our study further explored the effect of puncture to recanalization time on the favorable functional outcomes at 90 days and probability of death within 90 days in patients with progressive stroke, and found that prolonged puncture to recanalization time may negatively affect the prognosis and mortality of patients within 90 days. Actually, procedure time is related to several other factors, such as treatment experience, stroke types, site of occlusion, etc (25). Based on these effects, a more appropriate approach may be to evaluate each action on its own merits. Moreover, as we and others have shown EVT does not significantly increase the risk of surgery-related complications and adverse events.

## Limitation

This study has several limitations. First, our data are from multiple centers and may be subject to assessment bias. Second, the observational nature of our study, which has the inherent weaknesses of non-randomized clinical trials, such as selection bias in patient treatment. And our research is limited to the Chinese population and only reflects the situation in a specific area. It is necessary to conduct a larger multi-center study that includes different ethnic groups. Despite its limitations, it remains one of the best available data on the treatment of progressive stroke in acute BAO because it is a good representation of the daily clinical practice of patients with progressive stroke.

## Conclusions

Patients with progressive stroke in acute BAO can benefit from EVT. Compared with SMT, the EVT have improved efficacy and safety within 90 days. And EVT remains safe and effective in patients with progressive stroke when the treatment time window exceeds 6 h. In progressive stroke patients receiving EVT, lower baseline NHISS, higher baseline ASPECTs, and shorter PTR and recanalization are associated with a desirable outcome.

## Data Availability Statement

The original contributions presented in the study are included in the article/supplementary material, further inquiries can be directed to the corresponding authors.

## Ethics Statement

The study protocol was approved by the Ethics Committee of the Xinqiao Hospital, Army Medical University, in Chongqing, China, and each subcenter. The patients/participants provided their written informed consent to participate in this study.

## Author Contributions

YW, YK, and LW contributed to the conception and design of the study. ZL, YY, WL, and JY were responsible for approving the flow of related documents. XT, JL, WG, and DC wrote parts of the manuscript. YW and YK wrote the first draft of the manuscript. LW, QW, and JZ performed the statistical analysis. JS, ZQ, FL, and WZ organized the database. XW and ZY checked and revised the manuscript. All authors participated in the revision of the manuscript, read and approved the submitted version.

## Funding

This research was funded by the Sichuan Medical Association and Nanchong Science and Technology Bureau.

## Conflict of Interest

The authors declare that the research was conducted in the absence of any commercial or financial relationships that could be construed as a potential conflict of interest.

## Publisher's Note

All claims expressed in this article are solely those of the authors and do not necessarily represent those of their affiliated organizations, or those of the publisher, the editors and the reviewers. Any product that may be evaluated in this article, or claim that may be made by its manufacturer, is not guaranteed or endorsed by the publisher.
